# Impact of deleterious missense PRKCI variants on structural and functional dynamics of protein

**DOI:** 10.1038/s41598-022-07526-4

**Published:** 2022-03-08

**Authors:** Hania Shah, Khushbukhat Khan, Naila Khan, Yasmin Badshah, Naeem Mahmood Ashraf, Maria Shabbir

**Affiliations:** 1 Department of Healthcare Biotechnology, Atta-Ur-Rahman School of Applied Biosciences, National University of Sciences and Technology, Islamabad, Pakistan; 2 Department of Biochemistry and Biotechnology, University of Gujrat, Gujrat, Pakistan

**Keywords:** Biological techniques, Biotechnology, Cancer, Computational biology and bioinformatics

## Abstract

Protein kinase C iota (PKC_ɩ_) is a novel protein containing 596 amino acids and is also a member of atypical kinase family. The role of PKC_ɩ_ has been explored in neurodegenerative diseases, neuroblastoma, ovarian and pancreatic cancers. Single nucleotide polymorphisms (SNPs) have not been studied in PKC_ɩ_ till date. The purpose of the current study is to scrutinize the deleterious missense variants in PKC_ɩ_ and determine the effect of these variants on stability and dynamics of the protein. The structure of protein PKC_ɩ_ was predicted for the first time and post translational modifications were determined. Genetic variants of PKC_ɩ_ were retrieved from ENSEMBL and only missense variants were further analyzed because of its linkage with diseases. The pathogenicity of missense variants, effect on structure and function of protein, association with cancer and conservancy of the protein residues were determined through computational approaches. It is observed that C1 and the pseudo substrate region has the highest number of pathogenic SNPs. Variations in the kinase domain of the protein are predicted to alter overall phosphorylation of the protein. Molecular dynamic simulations predicted noteworthy change in structural and functional dynamics of the protein because of these variants. The study revealed that nine deleterious variants can possibly contribute to malfunctioning of the protein and can be associated with diseases. This can be useful in diagnostics and developing therapeutics for diseases related to these polymorphisms.

## Introduction

PKC_ɩ_ is a member of a serine threonine kinase family that plays an important role in the phosphorylation of hydroxyl group in protein residues (serine and threonine). The family has been categorized into three classes: Classical PKCs, Atypical PKCs and Novel PKCs. This grouping is based on the requirement of DAG and Ca^+2^ ions. Activation of classical PKCs require both DAG and Ca^+2^ ions while the Novel class needs only DAG for its functional activation. Atypical isoforms (PKC_ɩ_ and PKCζ) are different from the other PKC family members because of the distinct structural and functional characters. They do not need Ca^2+^ and diacylglycerol for the functional activation^1^. The studies show that the family of PKC is engaged in the causation of many diseases, for instance cancer, metabolic dysfunctions and cardiovascular disorders^2^.

PKC iota that has a significant role in the progression of cell cycle, its inhibition can lead to the obstruction of cell cycle progression. Lately, PKC_ι_ has also been studied in cancer cell line growth, metastasis and specific tumor gene amplifications^3^. One study demonstrated the role of PKC_ι_ in carcinogenesis through in vitro and in vivo studies^4^. The role of PKC in progression of cancer has been studied in later stages of cancer and metastasis. PKC isoforms in neoplastic diseases may transform into hyper or hypo activation^5^. Elevated expression of PKC_ɩ_ was noted in prostate, lung, ovarian and colon cancer^2^. PKC_ɩ_ is directly linked to oncogenic Ras signaling. It plays a significant role in Ras mediated transformation in intestinal epithelium that leads to malignancy in rats^4^.

The protein PKC_ι_ has been associated with numerous diseases but the link of its genetic variants with the diseases has not been established yet. For this purpose, the objective was to use various existing methods that predict the most deleterious missense variants in PKCι. Then, to use various bioinformatics tools on the predicted 3D structure of the protein to understand the possible impact of these variants on the structure and function of the protein, prediction of post translational modification of PKC_ι_ along with its involvement with cancer and survival. This is a first comprehensive in silico analysis of missense variants of this protein. The outcomes might be useful in designing precision medicines for associated diseases.

## Results

### Predicted structure of PKC_ι_ and post translational modifications

**Figure 1:**
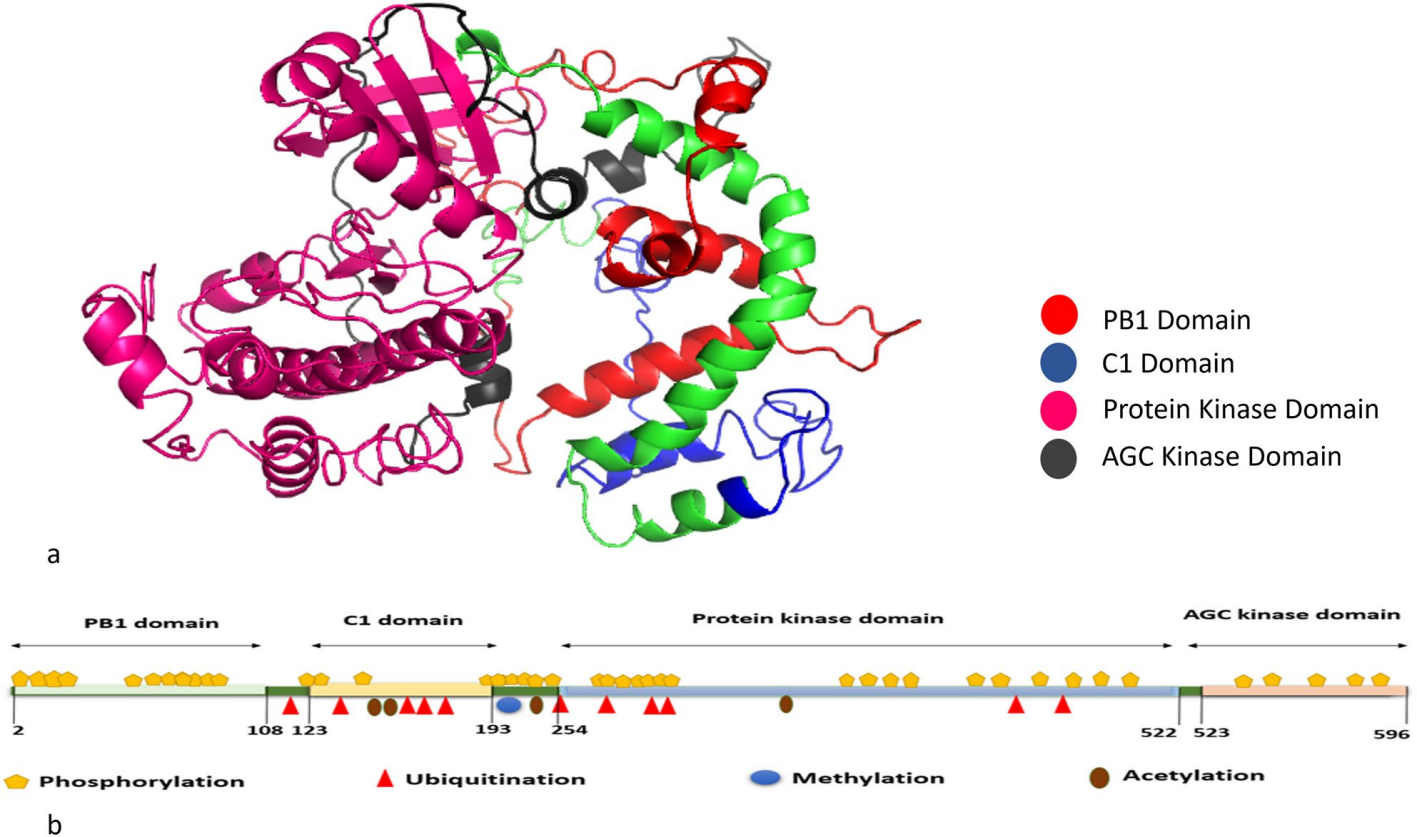
(**a**) Predicted Structure of Protein PKCι; It contains 4 domains. Red color represents PB1 domain (2–108 AA), C1 domain (123–192 AA) is highlighted in blue color, pink color shows Protein kinase domain (254–522) while dark grey color represents AGC kinase domain (523–596). (**b**) PKCɩ structure with predicted post translational modification sites distributed through its domains. Yellow pentagon shape is the representation of phosphorylation sites, red triangle is depicting ubiquitination sites, while blue circle is for methylation and acetylation is illustrated by brown oval shape.

Three dimensional (3D) structures of the protein were predicted via I-TASSER^6^ which is the most advance and reliable tool. It predicts protein structure based on multiple threading approach. Model 1 with a c-score of − 2.30 was selected^7^. The protein structure was predicted from I-TASSER because the complete structure with all important domains was not found in the protein structure data bank. The structure was then validated via INTERPRO^8^ and different domains of the protein were identified (Fig. 1a). Domains of the protein were highlighted through PYMOL. The protein is found to have 596 amino acids with four important domains: PB1 domain, C1 domain (Pseudo substrate domain), protein kinase domain containing the active site of the protein and AGC kinase domain. Alignment of PKC_**ι**_ structure was performed through PYMOL. Protein kinase domain of PKC_**ι**_ was aligned with crystal structure of kinase domain (38AX:ID from protein data base). An RMSD score of 0.944 was obtained. C1 and kinase domain of predicted structure were then aligned against solution structure of PKC-theta (1XJD, C1 and kinase domain). An RMSD score of 0.88 indicates that the structures are well aligned (Supplementary file 4, Fig. 1a,b).

Protein kinase domain is predicted to have maximum number of post translational modifications 17 phosphorylation sites (yellow pentagon), 6 ubiquitination sites (red triangles) and 1 acetylation (brown oval). AGC kinase and PB1 domain hosted only phosphorylation sites that were five and eleven in number respectively, while in C1 domain a total of three phosphorylation, four ubiquitination and two acetylation sites are observed. One methylation, one phosphorylation and a few phosphorylation sites were noticed in region 193–254 that comes in the regulatory domain (Fig. 1b) (Supplementary file 2; Data Tables 3, 4 and 5).

### Identification of variants in PKC_ɩ_ and calculation of %SNP effect

**Figure 2:**
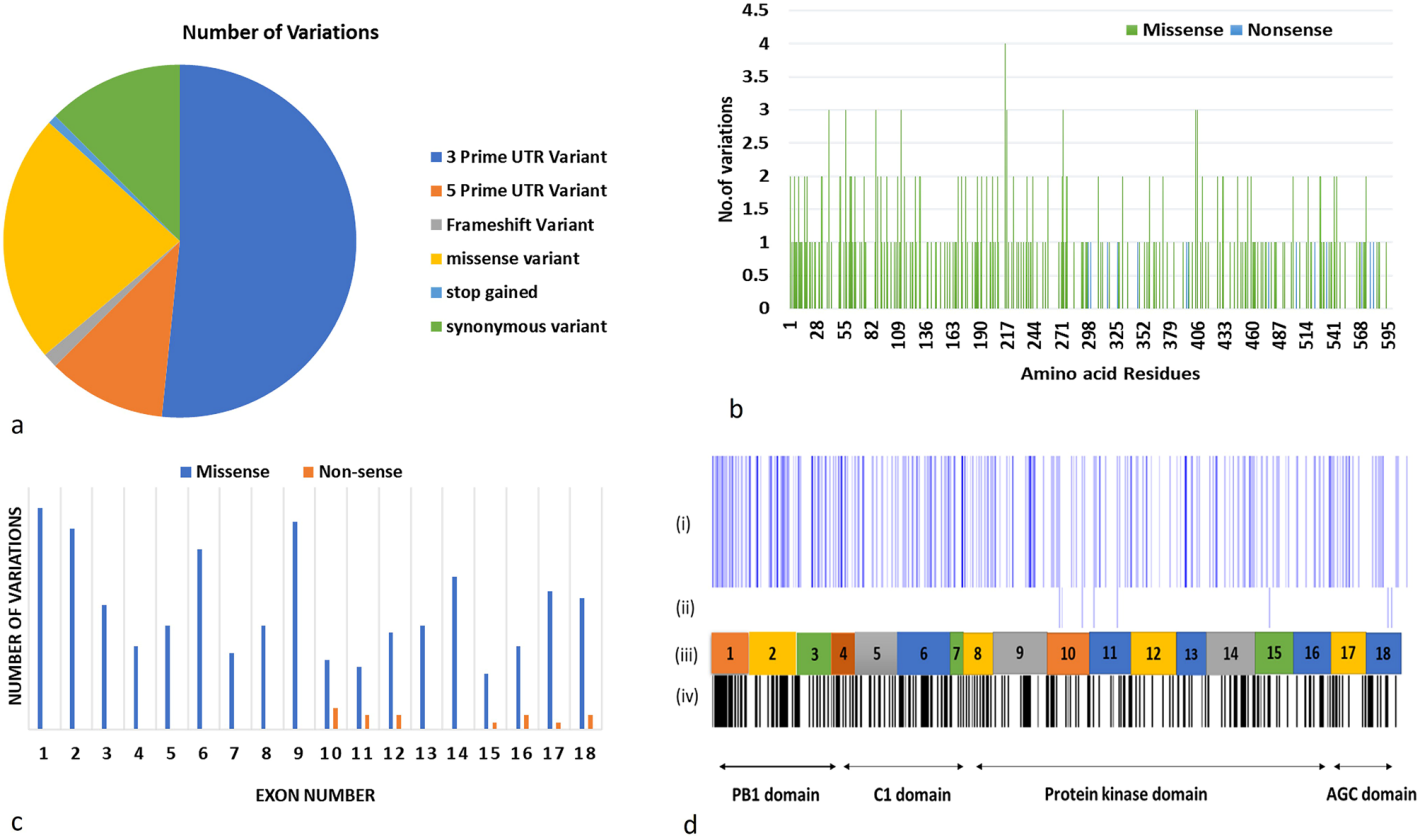
(**a**) Count of synonymous, stop gained, missense, frame shift, 5 and 3 prime UTR variants included in the study, (**b**) Mutational allocation of missense (green) and nonsense (blue) variation across protein co-ordinates of PKCɩ, (**c**) Distribution of missense (blue) and nonsense (orange) variations across 18 exons of PKCɩ, (**d**) Heat map drawn for missense SNPs (i), non-sense variations are illustrated in row (ii), while (iii) is the exons over which SNPs distribution are highlighted. Row (iv) is the null set of SNPs where probability of occurrence of SNPs is not high.

A total of 1317 SNPs of PKC_ɩ_ were collected from ENSEMBL data base (Fig. 2a). ENSEMBL data consists of variant information, protein functional annotations, disease association, and sequence data. The coding SNPs are found across 596 amino acid residues in PKC_ɩ_. Only missense SNPs (301) were selected for the further analysis, because mostly missense variants are found to be associated with diseases. A frequency of non-sense variants is very less as compared to missense variants and are concentrated in the Protein kinase and AGC kinase domain (Fig. 2b).

Exon wise relative abundance analysis of coding SNPs illustrated that exon one has the highest number of mutations (thirty-two in number), all of which are missense SNPs. Exon one encodes the PB1 domain of the protein. The lowest number of variations are displayed by fifteen exons containing a total of eight SNPs out of which seven are missense and one is non-sense. Exons that encode PB1 and C1 domain contained the highest number of variations. AGC domain has the lowest number of variations (Fig. 2c,d).

### Deleterious SNPs in PKC_ι_

**Figure 3:**
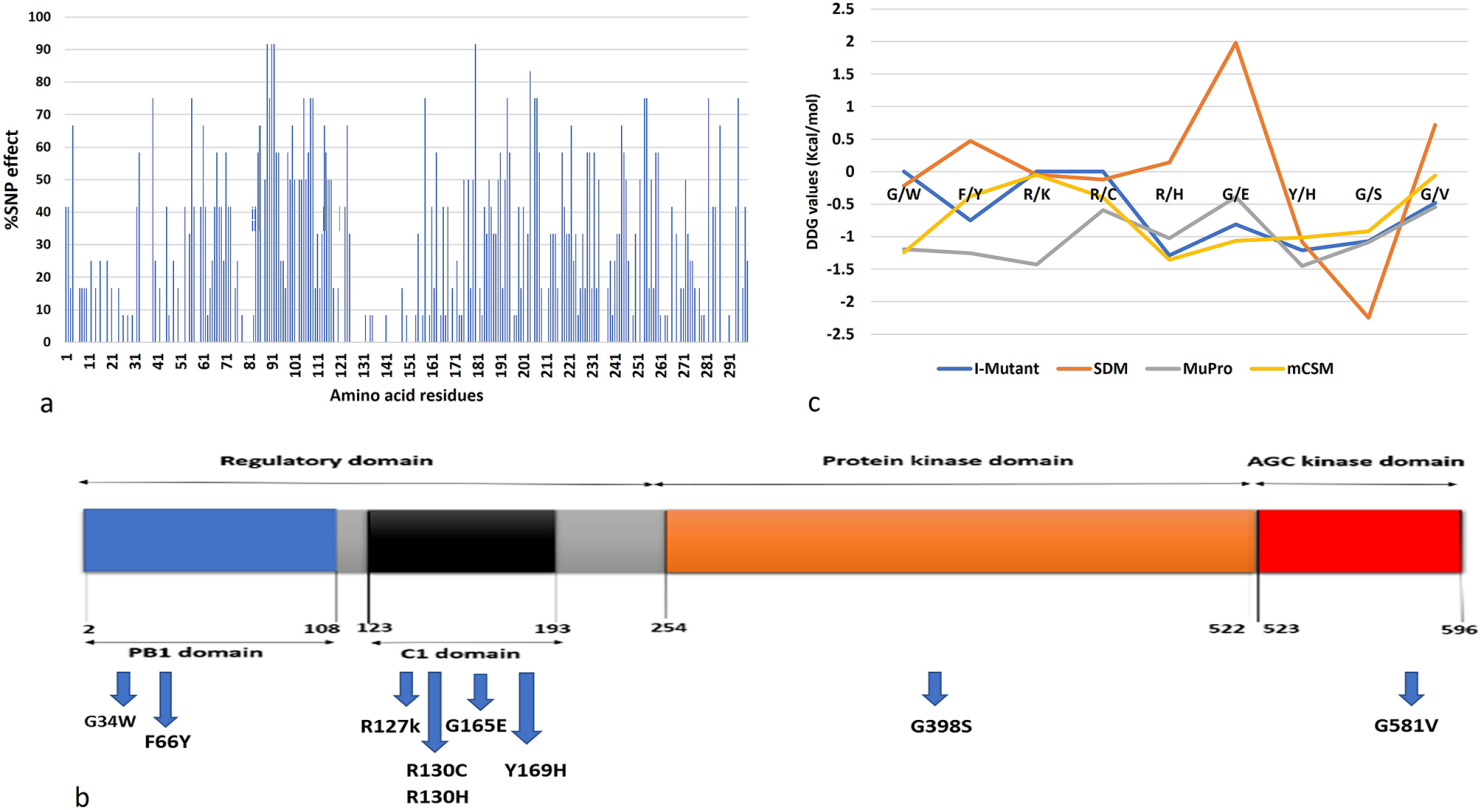
(**a**) Summary of percentage SNP effect of missense variations in each residue of the protein. (**b**) PKCɩ protein structure with domain wise distributed variants; PB1 domain (G34W, F66Y), C1 domain (R127K, R130C, R130H), Kinase domain of the protein (G398S) and AGC kinase domain (G581V). (**c**) Stability change DDG values of SNPs depicting destabilizing effect of selected SNPs.

The missense SNPs were analyzed on seven tools SIFT (≤ 0.05), POLYPHEN (> 0.9), REVEL (> 0.5), Mutation Accessor (≥ 0.8 = Medium, > 1.9 = high), CADD (≥ 30), MetaLR (> 0.5), and PROVEAN (≤ − 2.5), the cut off criterion for deleteriousness is shown in parenthesis (Supplementary file 1). The percentage SNP effect of missense variations were determined in each residue of the protein (Fig. 3a). The total number of identified SNPs varied in all the tools. SNP was considered as deleterious if pathogenicity was confirmed by > 75% tools (Table 1). After final scrutiny of deleterious and non-deleterious nine missense SNPs were identified to be highly deleterious with two SNPs residing in the PB1 domain, five in the C1 domain, one in the AGC domain while one in the protein kinase domain (Table 2) (Fig. 3b). The highest number of deleterious SNPs are found in C1 and pseudo kinase domain. This region is encoded by four, five, six, seven and eight exons. This region of the protein can be regarded as mutationally sensitive region of the protein. In atypical type of PKCs C1 domain is not responsive to DAG, it is catalytically activated by phospholipids that releases it from its membrane bound state to perform activities like wound healing, chemotaxis, and migration. The C1 domain can be targeted for drug development along with its hinge region^9^.

**Table 1:** Consolidated analysis scores of 9 selected SNPs through seven consensus tools.

Variant ID	SIFT	Polyphen	CADD	REVEL	MetaLR	Mutation Assessor	PROVEAN
rs1199520604	0	0.999	17	0.429	0.217	0.714	− 3.379
rs1197750201	0.03	0.999	28	0.588	0.539	0.729	− 3.542
rs146841636	0.03	0.996	29	0.582	0.756	0.807	− 6.781
rs56154494	0	0.983	31	0.62	0.688	0.825	− 4.191
rs369872734	0.02	0.983	32	0.694	0.751	0.825	− 4.247
rs1361108822	0.03	1	27	0.933	0.9	0.853	− 4
rs1050315708	0	0.942	27	0.891	0.91	0.927	− 6.172
rs773463648	0.02	1	28	0.841	0.76	0.89	− 3.747
rs1475798615	0	1	28	0.871	0.761	0.928	− 2.667

**Table 2:** Depicting variant IDs, genomic and amino acid co-ordinates along location of nine selected SNPs.

Variant ID	AA	AA coordinates	Location
rs1199520604	G/W	34	3:170222769
rs1197750201	F/Y	66	3:170235325
rs146841636	R/K	127	3:170267930
rs56154494	R/C	130	3:170267938
rs369872734	R/H	130	3:170267939
rs1361108822	G/E	165	3:170270464
rs1050315708	Y/H	169	3:170270475
rs773463648	G/S	398	3:170284585
rs1475798615	G/V	581	3:170303078

### Stability changes in mutant structures of protein

The effect of change in the stability of protein was predicted for nine selected SNPs. The stability prediction is based on DDG (delta delta G) values, it is metric of prediction of how single nucleotide polymorphism can affect the stability of a protein. It is the change in Gibbs free energy. A DDG score less than zero indicates lower stability. G34W, R127k, R130C, Y169H, and G398S are found to decrease the stability and F66Y, R130H, G165E, and G581V were found to destabilize the protein (Fig. 3c) (Supplementary file 3; Data Tables 1 and 2). Destabilization of a protein structure can alter its functional dynamics and can affect the normal pathways of the protein.

### Functional and physio-chemical analysis of selected SNPs

Project HOPE predicts the effects of Amino acid substitutions on the structural confirmations and functions of the protein. Project HOPE reveals some structural and functional changes in the protein because of these mutations that cause heritable diseases. Protein sequence and mutation are inserted as input query for HOPE. In case of 6 SNPs, the resultant residues are bigger than the wild one while in three SNPs the size has been reduced. This change can affect the overall structure and function of the protein. Most of the SNPs are in the regulatory region of the protein, making it mutationally sensitive region and affecting the regulatory function of the protein. In case of R130C, R130H and G165E the charge of the residue is also affected, changing from positive to neutral in case of R130C and R130H (Tables 3 and 4).

**Table 3:** Project HOPE analysis of deleterious SNPs in PB1 and Pseudo substrate region illustrating the changes in size, charge, hydrophobicity.

Residue	Structure	Properties
G34W		The mutant residue is bigger than the wild-type residue The mutant residue is more hydrophobic than the wild-type residue The mutation is located within a PB1 domain in Regulatory region
F66Y		The mutant residue is bigger than the wild-type residue The wild-type residue is more hydrophobic than the mutant residue The mutation is located within a PB1 domain in Regulatory region
R127K		The mutant residue is smaller than the wild-type residue The mutation is located within Pseudo substrate in regulatory region
R130C		The mutant residue is smaller than the wild-type residue The wild-type residue charge was POSITIVE, the mutant residue charge is NEUTRAL The mutant residue is more hydrophobic than the wild-type residue The mutation is located within Pseudo substrate in regulatory region
R130H		The mutant residue is smaller than the wild-type residue The wild-type residue charge was POSITIVE, the mutant residue charge is NEUTRAL The mutation is located within Pseudo substrate in regulatory region

**Table 4:** Project HOPE analysis of deleterious SNPs in the regulatory and Protein kinase domain illustrating the changes in size, charge, hydrophobicity.

Residue	Structure	Properties
G165E		The mutant residue is bigger than the wild-type residue The wild-type residue charge was NEUTRAL, the mutant residue charge is NEGATIVE The wild-type residue is more hydrophobic than the mutant residue The mutation is located regulatory region
Y169H		The mutant residue is smaller than the wild-type residue The wild-type residue is more hydrophobic than the mutant residue The mutation is located regulatory region
G398S		The mutant residue is bigger than the wild-type residue The mutation is located within Protein Kinase domain
G581V		The mutant residue is bigger than the wild-type residue The mutant residue is more hydrophobic than the wild-type residue The mutation is located within AGC-kinase C-terminal domain

### The most evolutionary conversed domain

**Figure 4:**
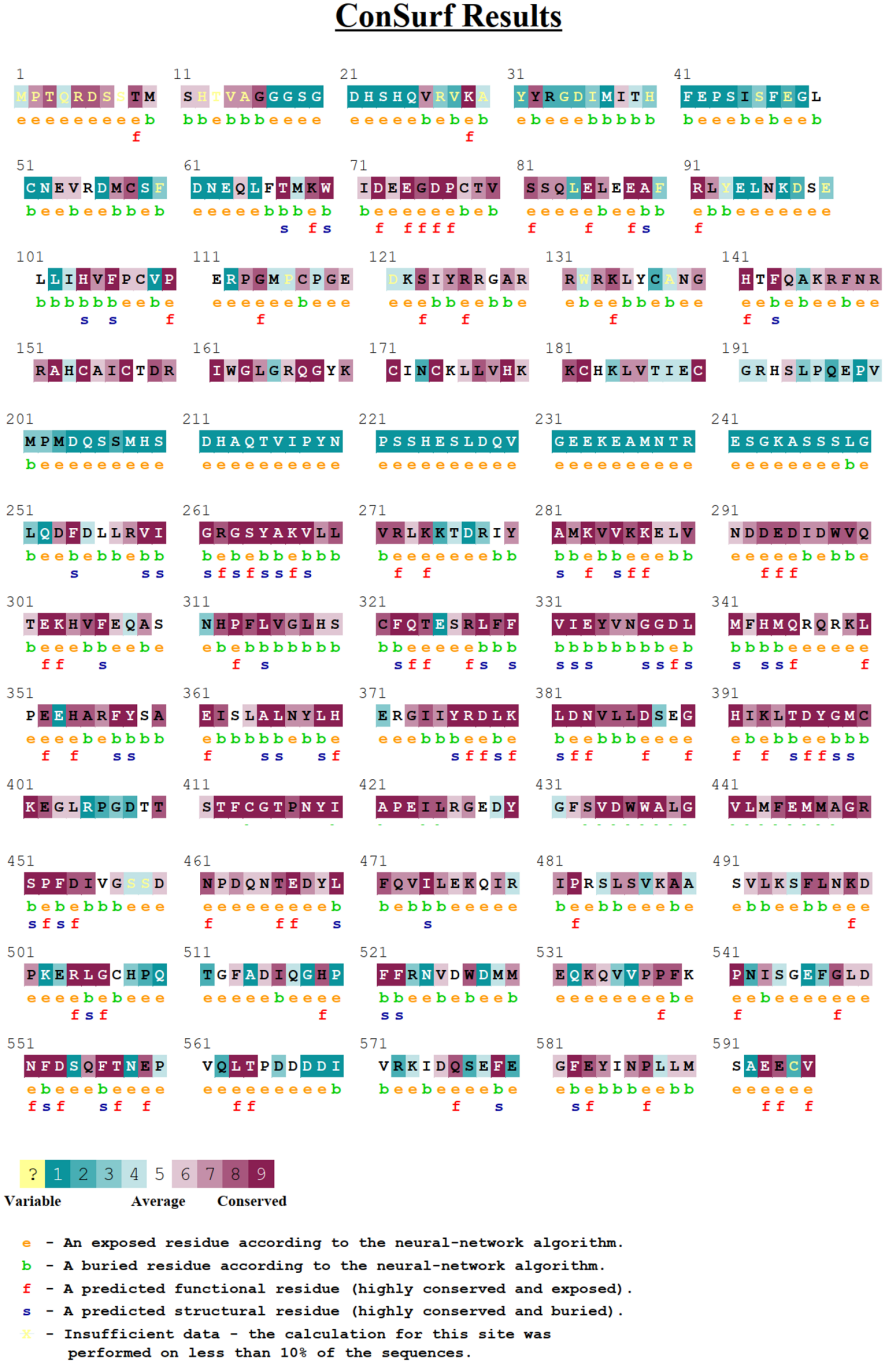
Protein conservation and surface accessibility analysis performed for 596 residues of protein PKCɩ on ConSurf tool.

According to ConSurf results for the PKC_ɩ_, the protein kinase domain is found to be evolutionary more conserved with a greater number of conserved amino acid residues. Literature also suggests that protein kinase domain is most conserved domain in PKC family members^10^. Few residues in the hinge region are conserved but its most residues were are variable. PB1 domain and pseudo substrate domain are found to be least conserved with very less evolutionary conserved residues (Fig. 4). Mutations in the conserved region of the protein are expected to be more damaging as compared to those in the less conserved region. Surface accessibility analysis gives an insight into the structure and function of Amino acid. The buried residues usually play a role in maintaining the structural integrity while the exposed residues are important for the protein- protein interactions. The SNPs G34W, R127K, R130C, R130H and G581V were found in exposed confirmations while F66Y, G165E, Y169H and G398S were found buried in the structure.

### Flexibility analysis of wild and mutant protein

**Figure 5:**
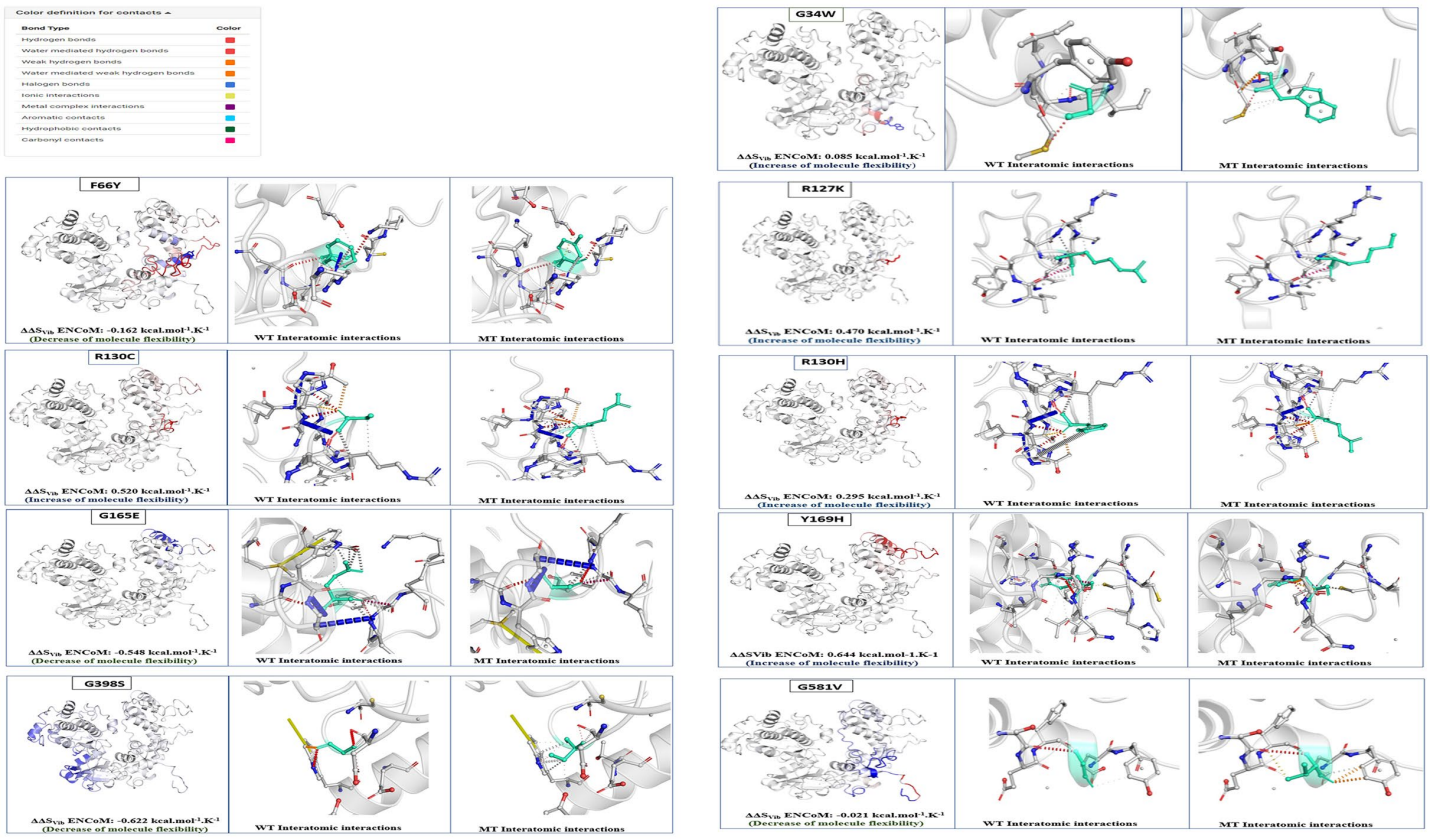
A comparison of molecular flexibility and destabilizing effect of mutants along with interatomic interactions in wild type and mutants by DynaMut tool.

The change in flexibility of the protein caused by nine SNPs was analyzed by DynaMut. The stabilizing effect was analyzed through ENcom values. According to the ENCom, values all of them are found to have destabilizing effect on the protein structure and function. Five variations (G34W, R127K, R130C, R130H, and Y169H) had increased molecular flexibility that was caused by increased vibrational entropy. Four variations (F66Y, G165E, G398S, G581V) were within the cut off value > 0.5 with decreased molecular flexibility. None of them has increased molecular rigidity. This change in the overall flexibility of the protein can affect the intramolecular interactions of the protein. A comparison of intramolecular interactions of wild and mutated structures is done in Fig. 5.

### Molecular dynamic simulations

Molecular dynamic simulations for nine SNPs were performed for 20 ns to get an insight into the confirmational changes in the protein structure due to missense mutations. The time scale of 20 ns is enough for the rearrangements of side chain in the wild structure and various parameters such as RMSF, RMSD, Radius of gyration, total number of intra-molecular hydrogen bonds, and SASA. Domain wise comparison of changes in mutants with wild structure was performed.

### Molecular dynamic simulation analysis in PB1 domain of the protein

**Figure 6:**
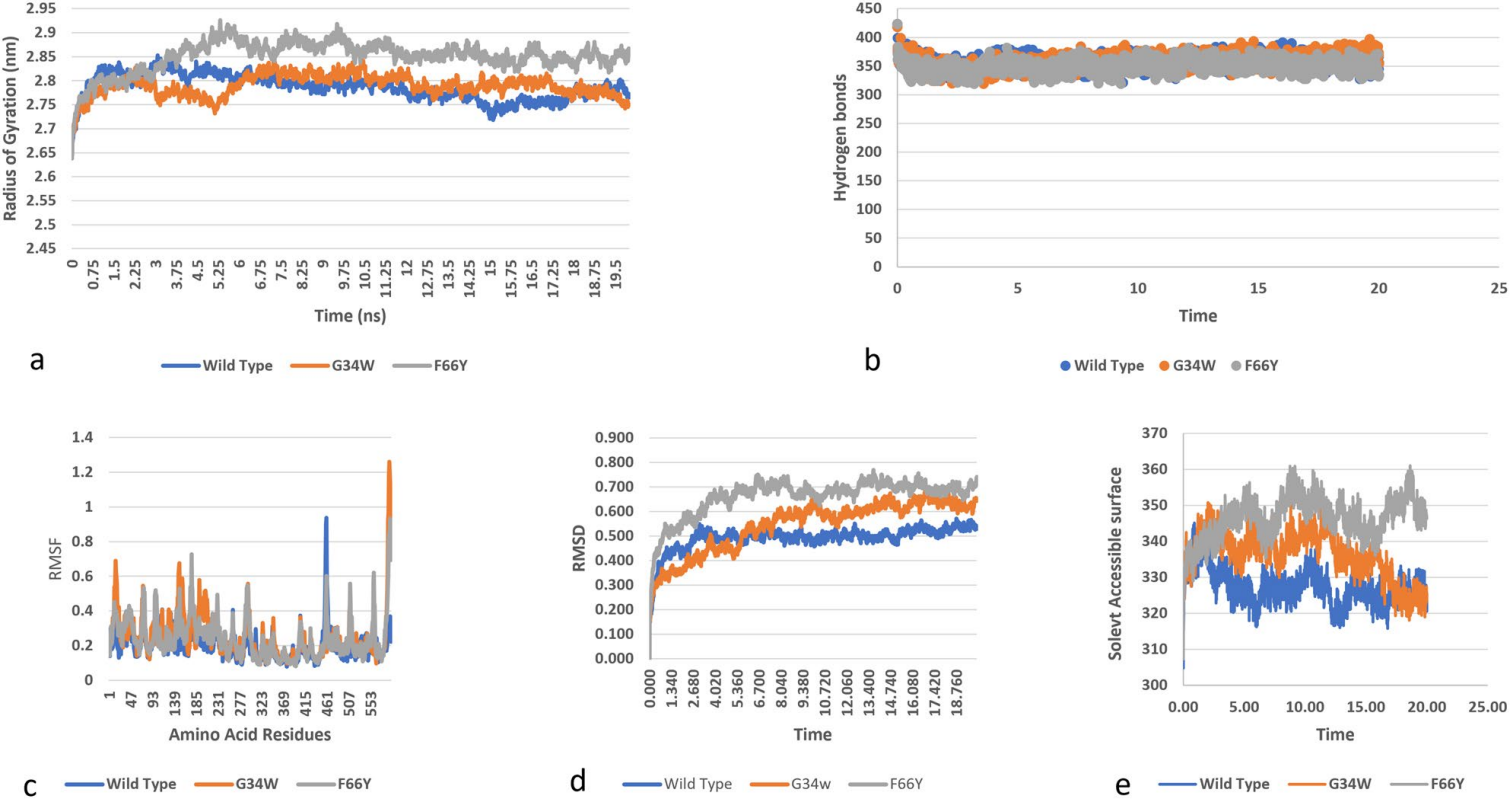
Simulation analysis of the missense SNPs in PB1 domain (G34W, F66Y) (**a**) Radius of gyration of the protein back bone (compactness of protein), (**b**) Total number of Hydrogen bonds throughout simulations of wild and mutant structure, (**c**) RMSF values of Carbon alpha in the simulation, (**d**) RMSD values of Cα atoms of wild and mutants, (**e**) Solvent accessible surface of wild and variants.

Two variants (G34W and F66Y) occupy the PB1 domain. The compactness of protein and mutants was examined by the radius of gyration. Wild protein has radius of gyration around 2.8 nm while highest gyration value of 2.87 nm is shown by F66Y at 3 ns. It is illustrated by the data that these structural destabilizations can lead to the loss of compactness to the protein structure as compared to the wild type PKC_ι_ (Fig. 6a). In wildtype protein as well in mutants, the total number of intramolecular hydrogen bonds contributes to the stability of the structure. Lowest number of hydrogen bonds are observed in F66Y around 310, followed by G34W having a mean of 320 H-bonds, with wild structure having around 400 bonds. The data suggest lower flexibility in structure with F66Y and G34W mutations (Fig. 6b).

For each residue of wild type and mutated protein fluctuations in RMSF were monitored to check the effect of mutation on dynamic behavior of protein residues. It is known from Fig. 6c that in G34W and F66Y the residue level fluctuations are quite high as compared to wild structure and other mutations. The wild protein has the highest fluctuation of 0.9 nm in residue 461. G34W has the highest fluctuation value of 1.4 nm in residue 576 while F66Y had the highest value of 0.9 nm in residue 580 residues (Fig. 6c). The effect of mutations on the structure of PKC_ɩ_ was analyzed by RMSD values. It is revealed from RMSD values that mutant structures are significantly unstable as compared to the wild structure (Fig. 6d).

It was showed that F66Y has higher SASA values followed by G34W. Both values are greater than wild structure. A higher SASA value indicates expansion of a protein, the results indicate that mutants are more unstable as compared to wild protein with F66Y being more unstable than G34W (Fig. 6e).

### Molecular dynamic simulations analysis of (C1 and pseudo substrate) regulatory domain of protein

**Figure 7:**
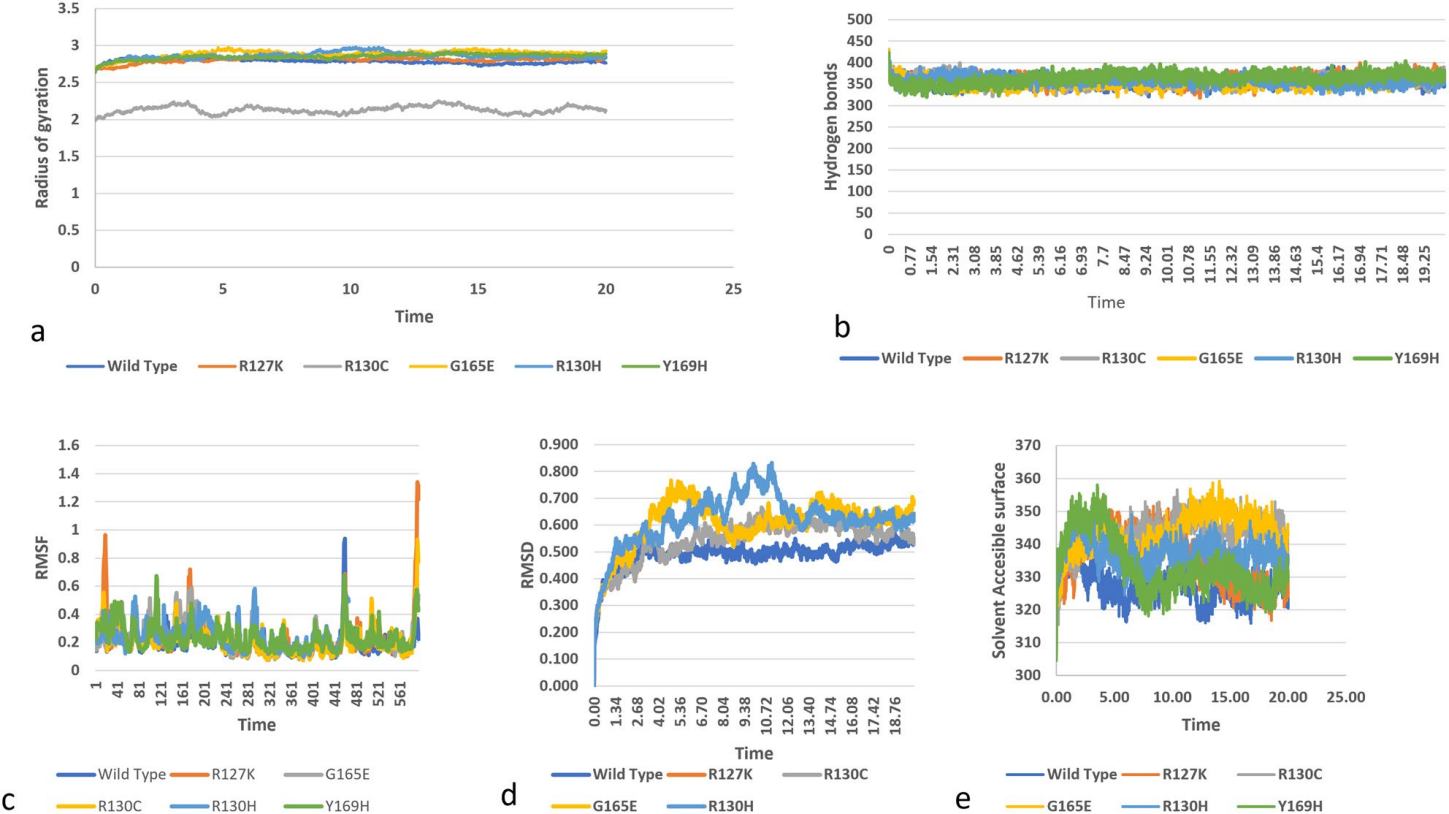
Simulation analysis of the missense SNPs in C1 domain (R127K. R130K, R130C, G165E, R130H, Y169H), (**a**) Radius of gyration of the protein back bone, (**b**) Total number of Hydrogen bonds throughout simulations of wild and mutant structure, (**c**) RMSF values of Carbon alpha in the simulation, (**d**) RMSD values of Cα atoms of wild and mutants, (**e**) Solvent accessible surface of wild and variants.

A total of 5 SNPs is observed in the C1 and pseudo substrate region. This region with PB1 and C1 domain make the regulatory region of the protein. In mutant R130C the radius of gyration has significantly reduced as compared to wild and other mutants, indicating a major change in the backbone of the protein structure, and altered compactness of the protein. Radius of gyration of other mutants is also changed (Fig. 7a). Maximum number of intramolecular hydrogen bonds in wild structure are around 400 while in mutants the number has been reduced. In Y169H lowest number of hydrogen bonds (an average of 360) are seen during 1–4 ns duration depicting decreased flexibility in its structure. Minor fluctuations in number of hydrogen bonds of other mutants are also observed (Fig. 7b).

Root mean square fluctuation (RMSF) values for each residue of native and mutant protein was examined. R127k had the highest RMSF of 1.3 nm at residues 561–596, followed by another fluctuation of 1 nm at residues 1–4. Y169H has the maximum fluctuation value of 0.7 nm from 441 to 481aminoacid residues. 0.9 nm fluctuation was recorded for R130C from 561 till last residues. G165E has a fluctuation of 0.5 nm from 161 to 201 residues. Maximum fluctuation of 0.6 nm was recorded for R130 from 281 to 290 residues (Fig. 7c). The effect of mutations on the structure of PKC_ι_ was analyzed by RMSD values. RMSD values showed that mutant structures are significantly unstable as compared to the wild structure (Fig. 7d). Maximum RMSD values were recorded for R130H, followed by G165E and then R130C. The difference between wild and R127K RMSD is not significant. It is demonstrated from the figure that mutation has considerable effect on the structure of PKC_ɩ_ (Fig. 7d).

From analysis solvent accessible surface area (SASA) it is exposed that Y169H has higher SASA values followed by G165E. After that R130H is found with higher values, with R127K values close to the wild structure. All mutant values are greater than the wild structure. A higher SASA value indicates expansion of a protein, the results indicate that mutants are more unstable as compared to wild protein with Y169H and G165E being more unstable than wild structure and other mutants (Fig. 7e).

### Molecular dynamic simulation analysis of protein kinase domain of protein

**Figure 8:**
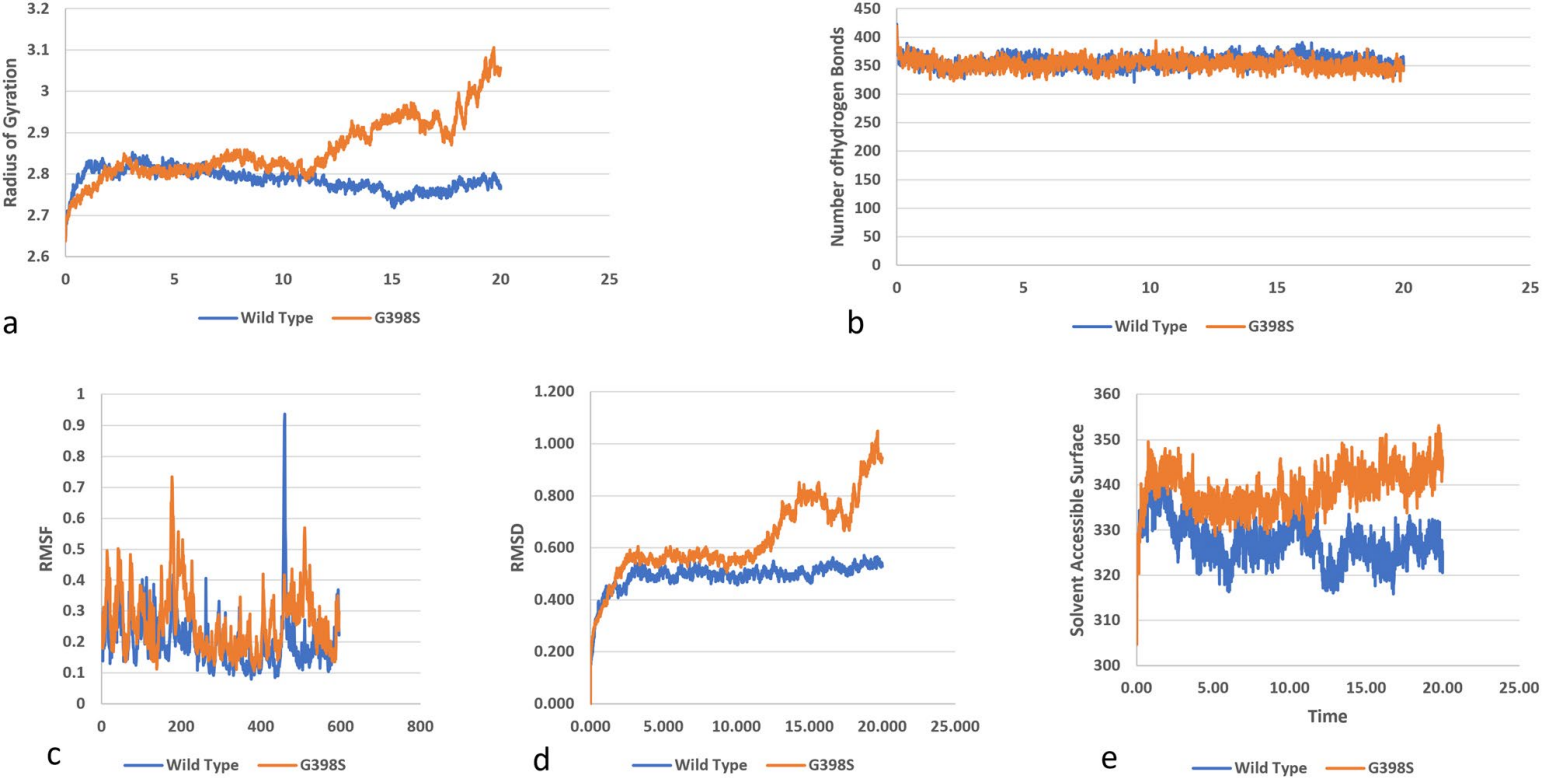
Simulation analysis of the missense SNP in Protein kinase domain (G398S), (**a**) Radius of gyration of the protein back bone, (**b**) Total number of Hydrogen bonds throughout simulations of wild and mutant structure, (**c**) RMSF values of Carbon alpha in the simulation, (**d**) RMSD values of Cα atoms of wild and mutants, (**e**) Solvent accessible surface of wild and variants.

SNP G398S is in the protein kinase domain of PKC_ɩ_. This domain is the most conserved domain of the family. The SNP is observed to cause alterations to the protein. The compactness of the protein is predicted to be majorly affected by this mutation (Fig. 8a). Radius of gyration has reached to a maximum of 3 nm during 20 ns duration. This is a huge increase as compared to the gyration values of wild protein (Fig. 8b). Overall number of hydrogen bonds in G398S have been reduced when seen in comparison to wild structure, depicting a decreased flexibility of structure (Fig. 8c).

In the root mean square fluctuation values, a noticeable change at each domain was observed. The highest RMSF peak of G398S was observed at 0.9 nm in residue 200 of the protein. Overall RMSF values of mutant were noticed to be higher as compared to wild protein. Root mean square deviation values were compared for wild and G398S mutant, a major change in stability was under observation depicting a highly unstable state of protein (Fig. 8d). From analysis solvent accessible surface area (SASA) it was illustrated that mutant G398S has higher SASA values than the wild structure. Indicating that the mutant is unstable as compared to wild protein (Fig. 8e).

### Molecular dynamic simulation analysis of AGC kinase domain of protein

**Figure 9:**
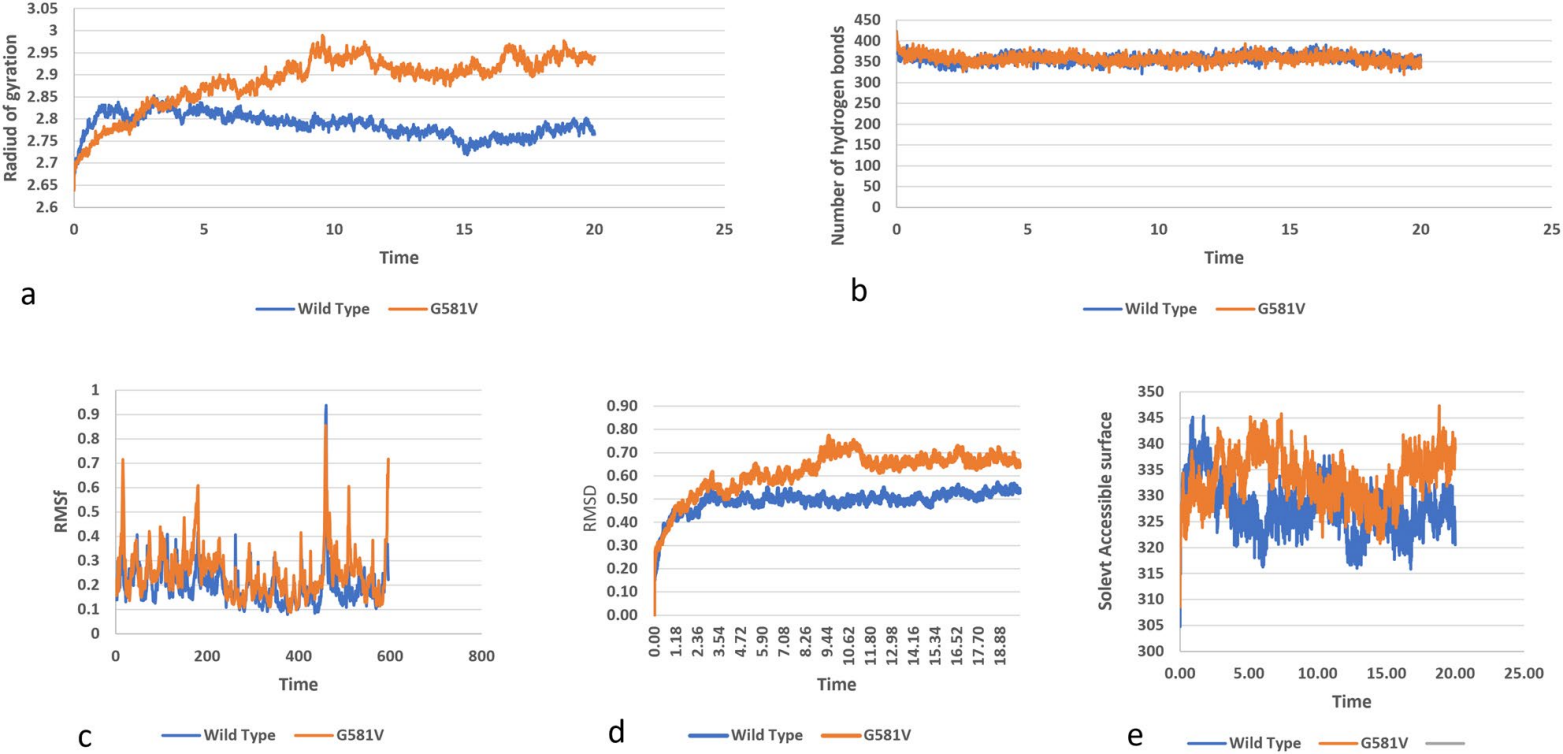
Simulation analysis of the missense SNP in AGC kinase domain (G581V), (**a**) Radius of gyration of the protein back bone, (**b**) Total number of Hydrogen bonds throughout simulations of wild and mutant structure, (**c**) RMSF values of Carbon alpha in the simulation. (**d**) RMSD values of Cα atoms of wild and mutants, (**e**) Solvent accessible surface of wild and variants.

Radius of gyration for mutant G581V is higher than the native protein and is increasing with the passage of time predicting a decrease in flexibility of the mutant structure (Fig. 9a). From hydrogen bond analysis wild structure was found to form more bonds than the mutant. The stability of mutant is therefore affected by fewer number of hydrogen bonds (Fig. 9b).

A significant difference in the RMSF values of wild and G581V was noticed. The highest peak of wild structure observed was 0.9 nm, while that of mutant was 0.8 nm at residue 500 of the protein (Fig. 9c). Other than that, the mutant peaks are at increasing trend when compared with peaks of wild structure. This imparts significant deviations in both structures. Also, RMSD values of G581V are higher than the wild structure (Fig. 9d). SASA analysis indicated that mutant has greater values than mutant. The reason for this change could be effect of substitution of amino acids by change in size of surface of protein (Fig. 9e).

### Association of pathogenic SNPs with cancer

The oncogenicity of selected SNPs were predicted through two tools. The FATHMM results for individual mutations are in the form of functional scores, SNP having score above 1 are considered as deleterious. CScape predicts the SNP as deleterious if the score is above 0.5. From FATHMM results, F66Y, G398S and G581V were predicted to be associated with cancer. CScape predicted all nine variants to be cancer drivers and oncogenic with a score greater than 0.6. Six variants F66Y, R127K, R130H, G165E, Y169H, G398S are categorized as high confidence oncogenic having score above 0.9.This suggests that all nine SNPs specifically F66Y, G398S and G581V can have possible role in protein dysregulation and causation of cancer (Table 5).

**Table 5:** Illustrating prediction of association of SNPs with cancer through FATHMM and CScape along with scores.

AA	AA coordinates	FATHMM prediction	FATMM score	CScape prediction	CScape score
G/W	34	Passenger	1.19	Oncogenic	0.630031
F/Y	66	Cancer	− 1.3	Oncogenic (high conf.)	0.904163
R/K	127	Passenger	0.67	Oncogenic (high conf.)	0.973039
R/C	130	Passenger	0.78	Oncogenic	0.829862
R/H	130	Passenger	0.64	Oncogenic (high conf.)	0.937806
G/E	165	Passenger	− 0.36	Oncogenic (high conf.)	0.951554
Y/H	169	Passenger	− 0.43	Oncogenic (high conf.)	0.911698
G/S	398	Cancer	− 1.65	Oncogenic (high conf.)	0.92553
G/V	581	Cancer	− 1.09	Oncogenic	0.880808

### Connection of PKC_ɩ_ with cancer through Kaplan–Meier plotter

**Figure 10:**
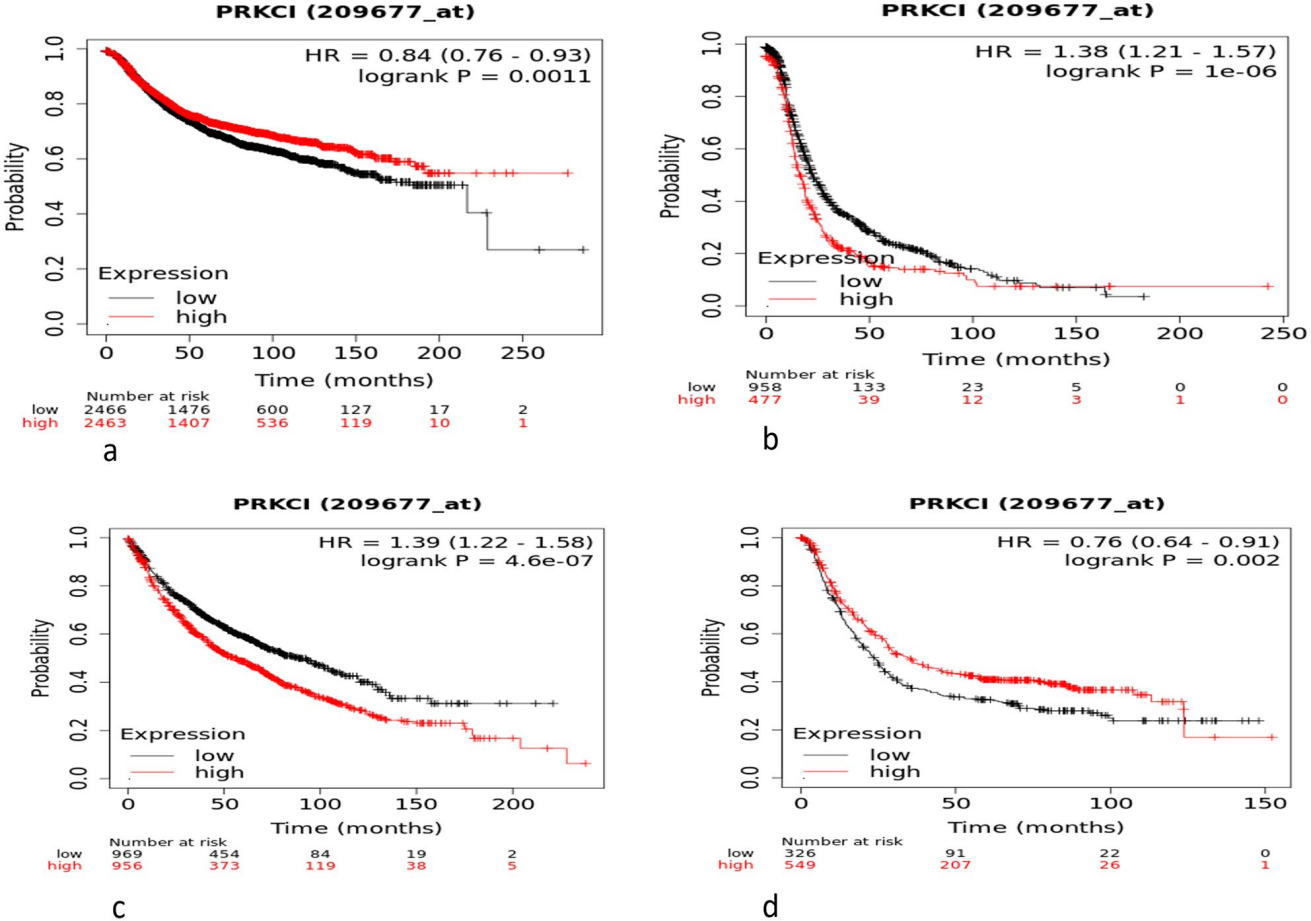
Probability survival curves for (**a**) Breast cancer, (**b**) Ovarian Cancer, (**c**) lung cancer, (**d**) Gastric cancer based on high and low expression of PKCɩ (Red line indicates higher expression of PKCɩ, while black line shows expression below the median line).

The effect of expression of PKC_ι_ on survival of cancer types like breast cancer, ovarian cancer, lung cancer and gastric cancer was determined through Kaplan–Meier Plotter. The red line is depicting the survival period of cancer patients having high expression levels of PKC_ι_, while the black line illustrates survival period of cancer patients with low expression levels of the protein (Fig. 10). This representation is in the form of Kaplan–Meier curve that shows probability of survival of patients at a certain time period.

Kaplan–Meier plotter analysis revealed that high and low expression of PKC_ɩ_ was found to have no significant link on survival of breast cancer, lung cancer, gastric cancer and Ovarian cancer patients. (Fig. 10a–d).

### Authentication of results through control study

For validation of our results, we performed a control analysis of the SNP, K274R which is proven as non-deleterious experimentally^11^. The score from Sift, Polyphen-2, CADD, MetaLR, PROVEAN, Mutation Assessor and REVEL predicted the SNP as non-deleterious, proving that these tools have a good accuracy level. The stability assessment of K274R was done through I-mutant, SDM, mCSM, MuPro and Dynamut. Except I-mutant & mCSM through which SNP is predicted as destabilizing, the other three tools prove it as stabilizing to the protein structure and function. Hope analysis also revealed that the SNP is possibly not damaging to the protein. Fathmm, and CScape both predicted the SNP to be benign. These results illustrated that all these tools have some accuracy level and can be used for filtration of deleterious SNPs that are to be tested experimentally (Supplementary file 3, Tables 1, 2, 3 and 4).

## Discussion

SNPs in the human genome can considerably affect characteristics and complex diseases through their regulation and modifications^12^. The data from literature advocated the role of single nucleotide polymorphisms in progression of several diseases. The genetic variants in PKC_ι_ are still unexplored, It is therefore vital to unravel the pathogenic SNPs in PKC_ι_ as these can directly affect the structure and role of a protein^13^. As missense variants are directly involved in pathogenicity and treatment regimen of a disease, that’s why only missense variants were considered for in-dept study^14^. The use of bioinformatic tools is an effective and cost-efficient method to analyze a large set of SNPs that are functionally important in a disease and can investigate mechanism and bases for these mutations^15^.

The protein structure was predicted and then aligned through PYMOL. The PKC_ɩ_ being a potent and multi-functional protein was characterized for non-sense and missense SNPs mutational hotspots present in the protein. An average of 15 variations per exon were estimated. SNPs in the protein kinase and AGC kinase domain were the lowest in number. The study focused on distribution of missense and non-sense SNPs variations on different residues, exons, and domains of the protein along with mutational sensitivity of the protein PKC_ɩ_ for these variations. Overall, Exon 1 contained the highest number of pathogenic and non-pathogenic SNPs, followed by exon nine and exon fifteen had the lowest number of SNPs. Exon one encodes PB1 domain, the PB1 domain is responsible for protein–protein interaction of PKC_ɩ_ with other protein having a PB1 domain such as MEK5/ERK (MAPK mitogen activated kinase/Extracellular signal regulated kinase) and Par-6 (partitioning-defective 6)^16,17^. The region or domain in which SNP is located has a great impact on protein. Dysregulation in expression is mostly because of SNPs in the regulatory region of the protein. Variants in PKC beta were reported to elevate insulin resistance in diabetic patients^18^. The most deleterious 9 SNPs G34W, F66Y, R130C, R127K, G165E, Y169H, G398S, R130H and 581V. were scrutinized that were D > 75% tools. C1 and the pseudo substrate domain contain the highest number of deleterious SNPs. C1 domain of PKC_ɩ_ is not dependent on DAG and Ca^+2^, its activity is enhanced by phospholipid phosphatidylserine^18^. The SNPs might affect the activation, resulting in altered behavior of the protein. The radius of gyration of R130C has been noticeably decreased as compared to wild and other mutants, increasing compactness of the protein. The rate of folding has a direct relationship with compactness of the protein^19^.

According to Project HOPE analysis in almost all the domains of the protein, amino acid substitutions have changed the size of the residues making it smaller or larger than the wild ones affecting the hydrophobicity and charge. This will disturb domain wise interaction. The difference in mass and charge leads to difference in protein–protein spatiotemporal dynamics^20^. The most conserved domain of the protein is protein kinase domain. It contained only one SNP out of the selected list. Kinase domain is homologous in all members of the PKC family^10^. The degree of conservancy was further confirmed by ConSurf tool. Maximum conversed regions were found in the kinase domain, which has important role in stabilizing the structure of protein (Ono et al.^10^). A noticeable change to the protein might be caused by these substitutions primarily affecting its stability that can misfold and change its function.

Through molecular dynamic simulations an insight into structural and functional dynamics of protein and mutants is exposed^21,22^. Many factors affect the process of protein folding, including conformational and compositional stability. Those factors include accessible surface area, packing density and residue depth. A mutation, changing an amino acid with another, may alter the conformation of the protein. Subsequently, the new structural rearrangement must affect the specific physio-chemical properties of the residue which is mutated. For determination of structure and function of a protein solvent accessibility (SASA) is a crucial factor, which is computed from sequences and structures of proteins through different algorithms. If the residue that is mutated is internal, the probability of unfolding and major changes increase, much less if the residue is superficial^23^. The protein cores consist of densely packed residues having a certain depth that maintains its packing density. This packing fraction can be perturbed if there is a change in the SASA values, this will lead to malfunctioned protein–protein interactions and membrane embedded portion of trans-membrane protein^24^. The variant F66Y in the PB1 domain is observed to increase SASA of the protein structure more as compared to other SNPs and wild type of the protein (Fig. 6). Consequently, decreasing the overall compactness and stability of the protein. This might be since F66Y is in buried residues of the protein (Fig. 4), The change in amino acid from tyrosine to Phenylalanine can disturb the overall interactions most probably PB1 domain interactions of the protein because of difference in the hydrophobicity of amino acids. The change in RMSD, radius and SASA of the SNP 398S suggested that this is destabilize the protein more as compared to other mutants. The reason behind this could be that the SNP is in the regulatory region of the protein. The protein kinase domain is responsible for the phosphorylation function of the protein, so the mutation can possibly affect the phosphorylation function of the protein. In protein interactions of PKC_ɩ_ with Par6 and Par3, protein kinase domain remains in closed confirmation^25^ the variant in the domain can possibly alter the protein interactions of PKC_ɩ_. Also, this is evident from Fig. 4 that G398S has a buried location in the protein, maybe that’s why its impact on protein functionality is substantial.

The pathogenicity and association of these 9 SNPs with cancer was confirmed through FATHMM and CScape. According to CScape all were oncogenic having 6 high confidence oncogenic SNPs with score above 0.9, the results from FATHMM demonstrated that F66Y, G398S and G581V are cancer related. These results are consistent with the result of MD simulations illustrating that these mutations can be significantly associated with cancer. The dysregulated expression of PKC_ɩ_ has been studied in various Ovarian Cancer^26^, Non-small lung carcinoma^27^, Colon Cancer^28^, Pancreatic Cancer^29^, Glioma^30^, Chronic myelogenous leukemia^31^ and Esophageal cancer^32^. But None of these variations were previously related with cancer. Variants in PKC beta were reported to elevate insulin resistance in diabetic patients^18^. Our studies of Kaplan–Meier plot illustrated that no significant association was found between expression of PKC_ɩ_ and breast, gastric, ovarian and lung cancer, however from literature it has been known that PKC_ɩ_ higher expression in gastric cancer can be linked with low overall survival^33^. PKC_ɩ_ expression in human non-small cell lung cancer (NSCLC) is over expressed and play an important role in altered growth of adenocarcinoma A549 human lung cancer cell line both in-vitro and in-vivo^27^. The study of control SNP revealed that in-silico tools can have some level of accuracy but as the computational tools used for scrutinization of SNPs are based on different algorithms, it is not necessary that highly conserved region variant always harvest noteworthy changes in the protein. Therefore, the confirmation of effects of these variants should be performed through genotype–phenotype based experiments. Generally, the study provides a starting point to investigate the deleterious variants in PKC_ι_ that can lead to altered structural dynamics mal function of the protein.

## Conclusion

PKC_ɩ_ as an oncogenic gene plays essential role in control of cell cycle and regulatory activities. Alteration in the expression of this gene can be associated with various diseases specifically cancer. The first comprehensive and systemic in-silico investigation of missense SNPs in the protein PKC_ɩ_ was performed. A total of 9 SNPs (G34W, F66Y, R127K, R130C, R130H, G165E, Y169H, G398S, G581V) were reported as potentially deleterious due to their capability of affecting protein stability and conformational dynamics. Domain wise post translational modifications study revealed that phosphorylation sites are concentrated at the protein kinase domain, this suggests that variant in protein kinase domain will strongly affect the phosphorylation strategy of the protein. Kaplan Meier Plotter suggested that high expression of PKC_ι_ can be associated with low survival rates. A connection of protein and the mutants with cancer was predicted, highlighting the fact that these can be used as important candidates in the prognosis and therapeutics strategies of cancer and other metabolic diseases.

## Methods

### Prediction of protein structure and post translational modification

The protein sequence of PRKCI gene with transcript ID: PRKCI-201 ENST00000295797.5 was obtained from ENSEMBL database in FASTA format. ENSEMBL incorporates data from more than 25 databases for homo sapiens that includes COSMIC, gnomeAD, ExAC, and dbSNP^34^.The data consists of variant information, protein functional annotations, disease association, and sequence data. The data comprise of genetic and disease specific studies. As the complete structure of PKC_ι_ is not found in PDB bank therefore this sequence was then submitted to I-TASSER (Iterative Threading Assembly Refinement)^35^ which is an online tool for prediction of protein structures based on the threading approach of protein modelling and generates each predicted protein model with a confidence score ranging from − 5 to 2^7,36^. The predicted models were then visualized with the help of PyMOL molecular visualization system. In addition, the predicted models by I-TASSER were cross-checked using InterPro database^8^ and other literature sources available, regarding the structural features of already studied and determined similar proteins. Validation of PKC_ι_ structure was performed by aligning kinase domain of the protein with crystal structure of PKC_ι_ (kinase domain, 38AX:ID from protein data base), similarly C1 and kinase domain of PKCɩ were aligned with PKC-theta ((1XJD, C1 and kinase domain).

Phosphorylation sites for PKC_ɩ_ was predicted through Netphos-GPS (http://www.cbs.dtu.dk/services/NetPhos/)^37^ with a cut-off score of 0.5. Values equal to and greater than 0.5 were considered. Methylation sites were predicted by GPS-MSP (http://msp.biocuckoo.org/)^38^, while GPS (pail) (http://pail.biocuckoo.org/)^39^ was used for acetylation sites. Ubiquitination was analyzed through PDM-PUB (http://bdmpub.biocuckoo.org/)^40^.

### Collection and processing of SNPs

Variations in the protein PKC_ɩ_ were identified from ENSEMBL (https://asia.ensembl.org/index.html)^41^. SNPs of PKC_ɩ_ excluding inframe and intronic were gathered and separated into regulatory variations (splice-site, 3′ and 5 UTRs) and coding SNPs (missense and non-sense SNPs). The data was retrieved in April 2021.The data base gave information about IDs of variants, amino acid coordinates, genomic coordinates, mutated base and amino acid residue information. Data about the protein was retrieved from Uniprot, InterPro and ENSEMBL. Residues of the protein were grouped into motifs, domains and loop regions. Within each domain and amino acid coordinate of the protein frequency of occurrence of SNPs was determined. Only missense variants were further subjected for prediction of pathogenicity. The scrutinized pathogenic SNPs were further mapped on exons and domains of the protein.

### Analysis of coding SNP effect

SNPs were analyzed on 7 tools for sorting intolerant from tolerant (SIFT)^42^, Polymorphism Phenotyping v2 (PolyPhen-2)^43^, Protein variation effect Analyzer (PROVEAN)^44^, Mutation Accessor^45^, Rare exome variant ensemble learner (REVEL)^46^, meta LR^47^ and Annotation dependent depletion (CADD)^48^. Through these tools, rigorous screening of deleterious SNPs was performed. SNP was considered deleterious only if more than 75% tools predicted it to be deleterious. Mutationally, the most sensitive region of PKC_ɩ_ was determined by taking average and percentage of the deleterious SNPs and then a total of nine SNPs were selected for final analysis of the study.

### Prediction of protein Stability

Stability change caused by the above mentioned mutations was determined through different tools such as mutation cut off scanning matrix (mCSM) (http://biosig.unimelb.edu.au/mcsm/)^49^, I-mutant (http://gpcr2.biocomp.unibo.it/cgi/predictors/I-Mutant3.0/I-Mutant3.0.cgi)^50^, Mu-Pro (http://mupro.proteomics.ics.uci.edu/)^51^ and Site directed Mutator (SDM) (http://marid.bioc.cam.ac.uk/sdm2)^52^. A variant was categorized as ‘Destabilizing’ SNP only when DDG values ≤ − 0.5 was given by at least 2 tools.

### Functional and physio-chemical analysis of selected SNPs

Functional analysis of the selected SNPs in protein gave an understanding of biological consequences of stability, sub cellular localization and membrane binding. The other physiochemical changes like size and charge of amino acid, hydrophobicity and involvement in hydrogen and salt bridge formation was drawn from project HOPE (https://www3.cmbi.umcn.nl/hope/)^53^.

### Evolutionary conservation study

Structure and function of the protein can be disrupted majorly by mutations that lie in the evolutionary conserved sections. The evolutionary conservation analysis of PKC_ι_ was performed through Consurf tool (https://consurf.tau.ac.il/) through conservation scores^54^.

### Flexibility analysis of selected variants

Effect of mutation on dynamics of a protein were assessed computationally through DynaMut (http://biosig.unimelb.edu.au/dynamut/), an online tool for predicting fluctuations in proteins through normal mode analysis^40^. The Elastic Network Contact Model (ENCoM) was considered as destabilizing if the score was DDG < − 0.5. Molecular flexibility was predicted to be increased if delta-vibrational entropy (DDS) > 0.5 while with a DDS < − 0.5 molecular flexibility was considered as decreased.

### Analysis of RMSD, RMSF, hydrogen bond and radius of gyration

The predicted model of PKC_ɩ_ was assessed for structural stability via GROMACS. Mutagenesis wizard tool of PyMOL^55^ was used to introduce point mutations. The obtained mutated structures were also examined for their influence on protein structure through GROMACS version 5.1. For stimulating the protein OPLS-AA force parameters were used^56^. The temperature was kept at 300 K while atmospheric pressure was maintained at 1. In a cubic box the system was solvated, neutralized and equilibrated for NVT and NPT simulation each. In detail ion steps are = 50,000, minim steps = 50,000, NPT steps = 50,000; 2 * 50,000 = 100 ps, NVT steps = 50,000; 2 * 50,000 = 100 ps and MD steps = 10,000,000; 2 * 10,000,000 = 20,000 ps (20 ns). MD simulation of 20 ns were performed on wild and mutated structures of the protein, the trajectory files were analyzed by Radius of gyration (Rg), Root mean Square Fluctuation (RMSF), Root mean square deviation (RMSD) and Solvent accessible surface (SASA).

### Association of PKC_ɩ_ and mutants with cancer

Pathogenicity of selected variations could have role in causation of different cancers. Association of pathogenic SNPs with cancer was predicted through tools CSCAPE (http://www.cscape.biocompute.org.uk/cgi-bin/submitcancer.cgi)^57^ and FATHMM (http://fathmm.biocompute.org.uk/)^58^. Through FATHMM the coding and non-coding variants were analyzed for its functional impact, while CScape was used for the prediction of oncogenic status of deleterious variants.

The effect of expression of PKC_ɩ_ on probability survival of different types of cancers such as breast cancer, ovarian cancer, lung cancer and gastric patients was determined through Kaplan–Meier Plotter, which is a software for integration of gene expression data with clinical data^59^. The data base contains information of over 22,277 genes and their impact on survival of breast, ovarian, lung and gastric cancer. The plot was generated and compared for survival of patients in low and high expression cohort.

### Authentication of results through control study

For assessment of our results through in-silico tools we took a control SNP, K274K from Uniprot. The SNP is in the kinase domain of the protein and has been proved to be non-deleterious to the structure and function of the domain and protein^11^. We applied Sift, PROVEAN, metaLR, CADD, Polyphen-2, REVEL and Mutation Assessor to K274K for pathogenicity test. Stability assessment was performed through SDM, MuPro, mCSM, Dynamut and I-mutant. Project Hope analysis for the SNP was also done. Prediction of cancer driver/passenger was checked through Fathmm and CScape.

## Supplementary Information

Supplementary Information 1.

Supplementary Information 2.

Supplementary Information 3.

Supplementary Information 4.
