# Supplementary material for: Impact of deleterious missense PRKCI variants on structural and functional dynamics of protein
Source: Sci Rep. 2022 Mar 8;12:3781. doi: 10.1038/s41598-022-07526-4 (PMC8904829; doi:10.1038/s41598-022-07526-4)
Supplement: Supplementary file 2 — Supplementary Information 2. [file 41598_2022_7526_MOESM2_ESM.pdf]

Table 1 illustrating DDG free energy of nine predicted by two tools I-mutant and SDM

SNP ID	AA substitution	AA coordinate	I-mutant (DDG change Kcal/mol)	stability	SDM(DDG change Kcal/mol)	stability
rs1199520604	G/W	34	-0.30	Decrease	-0.22	Decrease
rs1197750201	F/Y	66	-0.75	Increase	0.47	Increased
rs146841636	R/K	127	-1.09	Decrease	-0.05	Decrease
rs56154494	R/C	130	-1.08	Decrease	-0.12	Decrease
rs369872734	R/H	130	-1.29	Decrease	0.14	Increased
rs1361108822	G/E	165	-0.81	Decrease	1.98	Increased
rs1050315708	Y/H	169	-1.21	Decrease	-1.08	Decrease
rs773463648	G/S	398	-1.07	Decrease	-2.25	Decrease
rs1475798615	G/V	581	-0.48	Decrease	0.72	Increased

Table 2 depicting stability change through tools MuPro and mCSM

SNP ID	AA substitution	AA coordinate	Mu Pro (DDG change Kcal/mol	stability	mCSM (DDG change Kcal/mol)	stability
rs1199520604	G/W	34	Decrease	-1.192	Destabilizing	-1.246
rs1197750201	F/Y	66	Decrease	-1.2556	Destabilizing	-0.381
rs146841636	R/K	127	Decrease	-1.4297	Destabilizing	-0.052
rs56154494	R/C	130	Decrease	-0.5916	Destabilizing	-0.397
rs369872734	R/H	130	Decrease	-1.0247	Destabilizing	-1.356
rs1361108822	G/E	165	Decrease	-0.393	Destabilizing	-1.063
rs1050315708	Y/H	169	Decrease	-1.4492	Destabilizing	-1.014

rs773463648	G/S	398	Decrease	-1.0846	Destabilizing	-0.92
rs1475798615	G/V	581	Decrease	-0.5442	Destabilizing	-0.06

Netphos-3.1b prediction results

Table 3 Predicted phosphorylation sites of PKC-iota through Net-Phos

Sequence	# x	Context	Score	Kinase	Answer
# ---					
# PRKCI-201	3 T	--MPTQRDS	0.832	unsp	YES
# PRKCI-201	3 T	--MPTQRDS	0.698	PKC	YES
#					
# PRKCI-201	7 S	TQRDSSTMS	0.989	unsp	YES
# PRKCI-201	7 S	TQRDSSTMS	0.681	PKA	YES
#					
# PRKCI-201	8 S	QRDSSTMSH	0.996	unsp	YES
# PRKCI-201	8 S	QRDSSTMSH	0.602	PKC	YES
# PRKCI-201	8 S	QRDSSTMSH	0.567	PKA	YES
#					
# PRKCI-201	11 S	SSTMSHTVA	0.822	unsp	YES
# PRKCI-201	11 S	SSTMSHTVA	0.591	PKC	YES
#					
# PRKCI-201	39 T	DIMITHFEP	0.565	CKII	YES
# PRKCI-201	39 T	DIMITHFEP	0.506	cdc2	YES
#					
# PRKCI-201	44 S	HFEPSISFE	0.583	unsp	YES
#					
# PRKCI-201	46 S	EPSISFEGL	0.916	unsp	YES
# PRKCI-201	46 S	EPSISFEGL	0.504	CKI	YES
#					
# PRKCI-201	59 S	RDMCSFDNE	0.555	CKII	YES
#					
# PRKCI-201	67 T	EQLFTMKWI	0.812	PKC	YES
#					
# PRKCI-201	81 S	PCTVSSQLE	0.552	PKA	YES

#						
# PRKCI-201	82	S	CTVSSQLEL	0.717	unsp	YES
# PRKCI-201	82	S	CTVSSQLEL	0.612	CKII	YES
# PRKCI-201	82	S	CTVSSQLEL	0.584	ATM	YES
# PRKCI-201	82	S	CTVSSQLEL	0.576	DNAPK	YES
#						
# PRKCI-201	123	S	GEDKSIYRR	0.726	unsp	YES
#						
# PRKCI-201	125	Y	DKSIYRRGA	0.730	unsp	YES
# PRKCI-201	125	Y	DKSIYRRGA	0.535	EGFR	YES
#						
# PRKCI-201	158	T	CAICTDRIW	0.652	PKC	YES
#						
# PRKCI-201	194	S	CGRHSLPQE	0.951	unsp	YES
# PRKCI-201	194	S	CGRHSLPQE	0.839	PKA	YES
# PRKCI-201	194	S	CGRHSLPQE	0.546	DNAPK	YES
#						
# PRKCI-201	207	S	MDQSSMHSD	0.715	unsp	YES
#						
# PRKCI-201	210	S	SSMHSDHAQ	0.868	unsp	YES
#						
# PRKCI-201	215	T	DHAQTVIPY	0.594	unsp	YES
# PRKCI-201	215	T	DHAQTVIPY	0.536	PKC	YES
# PRKCI-201	215	T	DHAQTVIPY	0.513	cdc2	YES
#						
# PRKCI-201	222	S	PYNPSSHES	0.538	unsp	YES
# PRKCI-201	222	S	PYNPSSHES	0.508	CKII	YES
#						
# PRKCI-201	223	S	YNPSSHESL	0.991	unsp	YES
#						
# PRKCI-201	226	S	SSHESLDQV	0.568	CKI	YES
#						
# PRKCI-201	239	T	EAMNTRESG	0.545	cdc2	YES
#						
# PRKCI-201	242	S	NTRESGKAS	0.998	unsp	YES
# PRKCI-201	242	S	NTRESGKAS	0.905	PKC	YES
#						
# PRKCI-201	246	S	SGKASSSLG	0.673	PKA	YES

#						
# PRKCI-201	247	S	GKASSSLGL	0.536	cdc2	YES
#						
# PRKCI-201	248	S	KASSSLGLQ	0.790	unsp	YES
# PRKCI-201	248	S	KASSSLGLQ	0.588	cdc2	YES
# PRKCI-201	248	S	KASSSLGLQ	0.565	PKA	YES
#						
# PRKCI-201	264	S	IGRGSYAKV	0.783	PKA	YES
# PRKCI-201	264	S	IGRGSYAKV	0.773	PKC	YES
# PRKCI-201	264	S	IGRGSYAKV	0.570	DNAPK	YES
#						
# PRKCI-201	265	Y	GRGSYAKVL	0.518	INSR	YES
#						
# PRKCI-201	276	T	RLKKTDRIY	0.559	PKG	YES
#						
# PRKCI-201	301	T	DWVQTEKHV	0.986	unsp	YES
# PRKCI-201	301	T	DWVQTEKHV	0.521	CKII	YES
#						
# PRKCI-201	334	Y	FVIEYVNGG	0.902	unsp	YES
#						
# PRKCI-201	359	S	ARFYSAEIS	0.708	PKA	YES
#						
# PRKCI-201	363	S	SAEISLALN	0.554	PKA	YES
#						
# PRKCI-201	409	T	RPGDTTSTF	0.643	unsp	YES
#						
# PRKCI-201	411	S	GDTTSTFCG	0.506	cdc2	YES
#						
# PRKCI-201	412	T	DTTSTFCGT	0.888	unsp	YES
# PRKCI-201	412	T	DTTSTFCGT	0.552	PKC	YES
#						
# PRKCI-201	416	T	TFCGTPNYI	0.538	p38MAPK	YES
#						
# PRKCI-201	430	Y	RGEDYGFSV	0.789	unsp	YES
#						
# PRKCI-201	451	S	MAGRSPFDI	0.993	unsp	YES
# PRKCI-201	451	S	MAGRSPFDI	0.525	cdk5	YES
#						

# PRKCI-201	458	S	DIVGSSDNP	0.526	CKII	YES
# PRKCI-201	458	S	DIVGSSDNP	0.521	cdc2	YES
#						
# PRKCI-201	459	S	IVGSSDNPD	0.551	CKII	YES
#						
# PRKCI-201	466	T	PDQNTEDYL	0.554	CKII	YES
#						
# PRKCI-201	484	S	RIPRSLSVK	0.567	cdc2	YES
#						
# PRKCI-201	486	S	PRSLSVKAA	0.997	unsp	YES
# PRKCI-201	486	S	PRSLSVKAA	0.884	PKC	YES
# PRKCI-201	486	S	PRSLSVKAA	0.543	cdc2	YES
#						
# PRKCI-201	491	S	VKAASVLKS	0.900	unsp	YES
# PRKCI-201	491	S	VKAASVLKS	0.747	PKC	YES
#						
# PRKCI-201	495	S	SVLKSFLNK	0.658	PKC	YES
#						
# PRKCI-201	544	S	KPNISGEFG	0.945	unsp	YES
#						
# PRKCI-201	554	S	DNFDSQFTN	0.534	ATM	YES
#						
# PRKCI-201	564	T	PVQLTPDDD	0.974	unsp	YES
# PRKCI-201	564	T	PVQLTPDDD	0.515	CKII	YES
#						
# PRKCI-201	577	S	KIDQSEFEG	0.990	unsp	YES
# PRKCI-201	577	S	KIDQSEFEG	0.671	CKII	YES
#						
# PRKCI-201	584	Y	EGFEYINPL	0.947	unsp	YES
# PRKCI-201	584	Y	EGFEYINPL	0.561	EGFR	YES
# PRKCI-201	584	Y	EGFEYINPL	0.548	INSR	YES
#						
# PRKCI-201	591	S	PLLMSAEEC	0.727	unsp	YES
# PRKCI-201	591	S	PLLMSAEEC	0.518	CKII	YES

BDM PUB ubiquination

Table 4 showing Ubiquitination sites of PKC-iota through BDM-PUB

Peptide	Position	Score	Threshold
MPCPGEDKSIYRRGA	122	2.05	0.3
NGHTFQAKRFNRRAH	146	0.65	0.3
GYKCINCKLLVHKKC	175	0.96	0.3
NCKLLVHKKCHKLVT	180	1.84	0.3
LVHKKCHKLVTIECG	184	1.54	0.3
MNTRESGKASSSLGL	244	2.54	0.3
IGRGSYAKVLLVRLK	267	1.94	0.3
TDRIYAMKVVKKELV	283	1.23	0.3
YAMKVVKKELVNDDE	287	1.53	0.3
FHMQRQRKLPEEHAR	349	0.47	0.3
IPRSLSVKAASVLKS	488	2.53	0.3
VKAASVLKSFLNKDP	494	1.28	0.3

Table 5 illustrating acetylation sites through GSP-MSP

ID	Position	Peptide	Met-types	Score	Cutoff
PRKCI-201 peptide: ENSP00000295797 pep:protein_coding	240	EKEAMNTRESGKASS	R.di	4.03	3.87
