# Supplementary material for: Impact of deleterious missense PRKCI variants on structural and functional dynamics of protein
Source: Sci Rep. 2022 Mar 8;12:3781. doi: 10.1038/s41598-022-07526-4 (PMC8904829; doi:10.1038/s41598-022-07526-4)
Supplement: Supplementary file 3 — Supplementary Information 3. [file 41598_2022_7526_MOESM3_ESM.pdf]

Control Study for validity of Results

(SNP-K274R)

PATHOGENECITY

Table 1 illustrating deleteriousness of K274R through in-silico tools.

**Tools**	**Score**	**Deleteriousness**
Polyphen-2	0.070	Benign
PROVEAN	-2.409	Neutral
Sift	0.20	Tolerated
MetaLR	0.317	Tolerated
REVEL	0.28	Likely benign
Mutation Assessor	0.215	Low
CADD	21	Likely benign

STABILITY

Table 2 highlighting destabilizing effect of K274R through in-silico stability analysis tools.

**Tools**	**Score**	**Stability**
I Mutant	-0.61	Decreased
SDM	0.62	Increased
MuPro	-1.1330327	Decreased
mCSM	-0.674	Destabilizing
Dynamut	-0.358	Destabilizing

PROJECT HOPE

Table 3 depicting structural impact of K274R on protein through Project Hope.

**K274R**	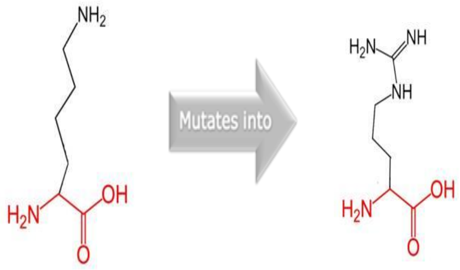	• The mutant residue is bigger than the wild-type residue.<br>• The mutation is located within a Protein Kinase domain.<br>• Mutagenesis experiments have been performed on this position and shows no effect on activity.<br>• This mutation is possibly not damaging to the protein

CANCER ASSOCIATION

Table 4 illustrating association of K274R with cancer through Fathmm and CScape.

**Tools**	**Substitution**	**AA coord**	**prediction**	**Score**
Fathmm	K274R	274	Passenger/Other	0.84
CScape	K274R	274	Benign	0.47
