# Supplementary material for: Impact of deleterious missense PRKCI variants on structural and functional dynamics of protein
Source: Sci Rep. 2022 Mar 8;12:3781. doi: 10.1038/s41598-022-07526-4 (PMC8904829; doi:10.1038/s41598-022-07526-4)
Supplement: Supplementary file 4 — Supplementary Information 4. [file 41598_2022_7526_MOESM4_ESM.docx]

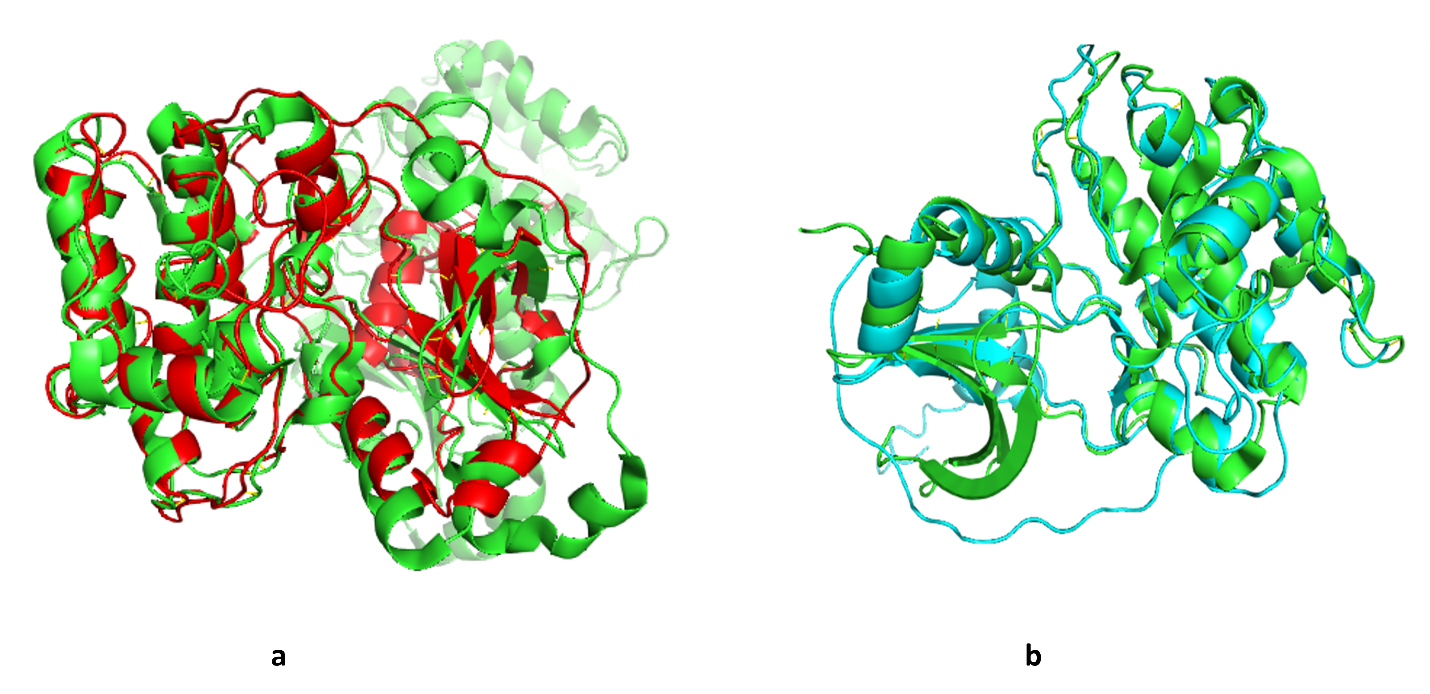


**Figure 1: (a) Alignment of Predicted structure of PKCɩ (i-TASSER, Kinase domain) with crystal structure of kinase domain of PKCɩ (38AX:ID from protein data base). (b) Alignment of Predicted structure of PKCɩ (i-TASSER, C1 &Kinase domain) with crystal structure of C1 & kinase domain of PKC-theta (1XJD:ID from protein data base).**
